# Efficacy of chlorine dioxide mouthwash in reducing oral malodor: A 2‐week randomized, double‐blind, crossover study

**DOI:** 10.1002/cre2.131

**Published:** 2018-10-23

**Authors:** Thuy Anh Vu Pham, Ngoc Thi Xuan Nguyen

**Affiliations:** ^1^ Department of Periodontology, Faculty of Odonto‐Stomatology University of Medicine and Pharmacy Vietnam; ^2^ Faculty of Odonto‐Stomatology University of Medicine and Pharmacy Vietnam

**Keywords:** chlorine dioxide mouthwash, Gram negative bacteria, Gram positive bacteria, oral malodor

## Abstract

The aim of this clinical trial was to assess the inhibitory effects of a mouthwash containing 0.1% ClO_2_ used for 2 weeks on oral malodor, periodontal and salivary parameters, tongue coating, and Gram‐negative and Gram‐positive bacteria in saliva. Thirty‐nine subjects with oral malodor were randomly assigned into two groups. In the first stage, one group was instructed to rinse with the experimental mouthwash (containing 0.1% ClO_2_), whereas the other group was instructed to rinse with the control mouthwash (sodium chloride 0.9%) for 2 weeks. After 4 weeks of washing out, in the second stage, each group then used the other mouthwash for 2 weeks. Oral malodor, periodontal status, tongue coating, salivary pH and flow rate, and the amounts of the salivary bacteria *Aggregatibacter actinomycetemcomitans*, *Fusobacterium nucleatum*, *Porphyromonas gingivalis*, *Solobacterium moorei*, *Streptococcus salivarius*, *Treponema denticola*, and *Tannerella forsythia* were evaluated at baseline and after 2 weeks of mouthwash use. After 12 hr and after 2 weeks, organoleptic scores and the levels of H_2_S and CH_3_SH were significantly lower in the experimental group compared with those in the control group. After 2 weeks, the experimental mouthwash appeared significantly effective in reducing plaque index, tongue‐coating score, and the amounts of *F*. *nucleatum*, S. moorei, *T*. *denticola*, and *T*. *forsythia* in the whole saliva, compared with those at baseline. Mouthwash containing 0.1% ClO_2_ is effective in reducing oral malodor, dental plaque, tongue‐coating accumulation, and the amounts of *F*. *nucleatum*, S. moorei, *T*. *denticola*, and *T*. *forsythia* in saliva.

## 1 INTRODUCTION

Oral malodor, commonly known as halitosis or bad breath, describes an offensive odor that emerges from the oral cavity and is easily detected by others (Tonzetich, 1977). Oral malodor is not a serious illness, but it deeply affects communication and the psychological issues of patients. It is also one of the leading causes for patients to seek dental treatment, just behind caries and periodontal disease (Loesche & Kazor, 2002).

The origins of oral malodor mainly come from various products of bacterial metabolism, such as amino acids, oral epithelial cells, and white blood cells. The products of this metabolism include volatile sulphur compounds (VSCs), indoles, skatoles, amines, and ammonia. VSCs are mainly hydrogen sulphide (H_2_S), methyl mercaptan (CH_3_SH), and dimethyl sulphur (CH_3_)_2_S, which are thought to be the main cause of oral malodor (Tonzetich, 1977). Some studies have demonstrated that a variety of periodontal bacteria, including *Aggregatibacter actinomycetemcomitans*, *Porphyromonas gingivalis*, *Fusobacterium nucleatum*, *Tannerella forsythia*, and *Treponema denticola*, play an important role in the production of VSCs and are closely associated with oral malodor (Awano, Gohara, Kurihara, Ansai, & Takehara, 2002; Nakano, Yoshimura, & Koga, 2002; Persson, Edlund, Claesson, & Carlsson, 1990). However, these oral anaerobic bacteria may yield varying amounts and ratios of VSCs.

Although Gram‐negative bacteria have been recognized as a major contributor to oral malodor, many studies have shown that Gram‐positive microorganisms also play an important role (Haraszthy et al., 2008; Stephen, Naughton, Pizzey, Bradshaw, & Burnett, 2014; Vancauwenberghe et al., 2013). Although Gram‐positive bacteria are weak producers of VSCs, they are responsible for the deglycosylation step, which is required for the subsequent degradation of some proteins by Gram‐negative bacteria (Sterer & Rosenberg, 2006). Moreover, recent studies have also shown that some Gram‐positive bacteria such as *Solobacterium moorei* and *Streptococcus salivarius* may also be involved in the production of odorous compounds (Sterer & Rosenberg, 2006; Vancauwenberghe et al., 2013). There have been many conflicts about whether a specific flora associated with oral malodor exists and which species are highly correlated with the presence of halitosis.

Developing an elimination method that targets various halitosis‐causing bacteria and controls tongue coating remains a challenge. Some antibacterial agents have been introduced to reduce or inhibit halitosis bacteria. Chlorine dioxide (ClO_2_) is an oxidant compound that is widely used in many fields. A number of clinical studies have shown that ClO_2_ decreases the amount of VSCs by oxidating amino acids such as cysteine and methionine, which are substrates of malodorous substances (Grootveld, Silwood, Gill, & Lynch, 2001; Lynch et al., 1997). There have been some studies investigating the impact of ClO_2_ mouthwash on oral malodor status; however, most of them were carried out on people with healthy breath and rarely assessed the microbiology results, especially for Gram‐positive organisms (Frascella, Gilbert, Fernandez, & Hendler, 2000; Peruzzo, Jandiroba, & Filho, 2007; Shinada et al., 2010; Shinada, Ueno, Konishi, Takehara, & Yokoyama, 2008; Soares, Guaitolini, Weyne Sde, & Falabella, 2013). Therefore, the aims of this study were to evaluate the effect of 0.1% ClO_2_ mouthwash on halitosis patients, considering oral malodor, periodontal and salivary parameters, tongue coating, and the salivary bacteria *A*. *actinomycetemcomitans*, *F*. *nucleatum*, *P*. *gingivalis*, S. moorei, *S*. *salivarius*, *T*. *denticola*, and *T*. *forsythia* for 2 weeks.

## 2 MATERIALS AND METHODS

### 2.1 Subjects

A total of 62 first‐year to third‐year students at the University of Medicine and Pharmacy, Ho Chi Minh City, who had halitosis as a chief complaint were voluntarily participated for the study. The individuals were then screened; to be included, 51 students had to present with (one) an organoleptic score ≥ 2 based on the Rosenberg scale (Rosenberg & McCulloch, 1992), (two) a level of H_2_S > 1.5 ng/10 ml, or CH_3_SH > 0.5 ng/10 ml.^1^ Students who suffered from systemic diseases (e.g., diabetes mellitus, gastrointestinal disorder, respiratory dysfunction, neoplasia, and various carcinoma), who took medication or antibacterial substances, conditions known as causes of extraoral halitosis, who were wearing dentures or orthodontic appliances, and who underwent any antibiotic treatment 1 month before and during the study course were excluded. Forty participants fulfilled the criteria and were selected into the study. All participants were provided with information regarding risks and benefit of the study, and written informed consent was obtained. During the study, one subject was eliminated because they did not participate in the full protocol, so that the final sample was 39 participants (19 men and 20 women). The study was approved by the Ethical Committee of the University of Medicine and Pharmacy, Ho Chi Minh City (reference number: 16142‐ĐHYD, 186/ĐHYD‐HĐ), and was performed between October and December 2016 at the Faculty of Odonto‐Stomatology. This trial has been conducted in full accordance with the World Medical Association Declaration of Helsinki. It also was registered and listed on the Standard Randomized Controlled Trial Number (ISRCTN) registry with study ID: ISRCTN75902618. Before the study was conducted, the necessary sample size of 18 participants was calculated for each group using the formula $N=R\left(\frac{\;2{\left[z_{1-\alpha /2}+z_{1-\beta }\right]}^{2}}{\Delta^{2}}+\frac{z_{1-\alpha /2}^{2}\;}{4}\right)$ with *R =* $\;\left(\frac{\;1+\left[w-1\right]\rho }{w}-\frac{v\rho^{2}\;}{\left[1+\left(v-1\right)\rho \right]}\right)$; *Z*_1 − α/2_ = 1.96, level of significance *α* = 0.05; Z_1−*β*_ = 0.84, power *β* = 0.2; Δ = 0.53, expected difference in the reduction of organoleptic measurement score between two mouthwashes (Shinada et al., 2010); *p* = 0.75, correlation coefficient of the repeated measurements; *v* = 1, the number of measurements before treatment; *w* = 2, the number of measurements after treatment. The calculated sample size for each group was 18. With the hypothesis that a 10% of all participants would be lost to follow up or drop out of the study, we have determined that there would be 20 participants needed for each group.

### 2.2 Materials

The experimental sample was commercial mouthwash (TheraBreath® Mild Mint Oral Rinse) containing 0.1% chlorine dioxide. The control sample was 0.9% sodium chloride solution with additional flavors to imitate the taste of the experimental oral rinse. Both mouth rinse samples (experimental and control) were put into identical white opaque plastic bottles. An independent person, outside this study, labelled the bottles with code A or B for experimental or control mouthwash. Neither examiners nor subjects in the research group knew, which were the experimental or control samples until the study was completed.

### 2.3 Study design

This study was a crossover, randomized, double‐blinded clinical trial with a 4‐week washout period between two 2‐week stages. The subjects were randomized into two groups by a person (2‐year graduate student) who was outside the trial. This assignment was secured secretly in the patient records and only revealed (if necessary) after the trial ended. The graduate student has also instructed participants how to rinse the mouthwashes in this trial.

In the first stage, one group was instructed to rinse with 30 ml of the experimental mouthwash twice daily (morning and evening) for 2 weeks, whereas the other was instructed to rinse with 30 ml of the control mouthwash in the same way. Participants were instructed to use their mouthwash in the following way: Rinse with 15‐ml mouthwash for 30 s, then spit and continue to gargle with 15‐ml mouthwash for 15 s. After 4 weeks of washout, in the second stage, each group used the other mouthwash for 2 weeks. During the study, participants were given dentifrice (P/S Cavity Fighter, Unilever, Vietnam) to use and continued to brush their teeth in their own way. They were asked to stop the mouthwash if they had any problem such as allergy or vomiting. They were also not allowed to rinse with other kinds of mouthwash nor brush their tongues.

### 2.4 Oral malodor assessments

Oral malodor was measured by an organoleptic measurement, and the amount of VSCs was analyzed by OralChroma™ Model CHM‐1 (Abimedical, Abilit Corp., Osaka, Japan) around 9 a.m. Participants were instructed to abstain from eating food with a strong smell for 48 hr, from drinking alcohol or beer, and from using perfumes or fragranced cosmetics for 12 hr before the assessment. Besides that, they were advised not to practice their oral hygiene practice nor to ingest any food or drink on the morning of the assessment day. Participants were frequently contacted via telephone or social network by investigators to remind them of regular rinsing. In addition, at each follow‐up appointment, participants were required to bring the given bottles, to assess their compliance at home through the amount of mouthwash remaining.

#### 2.4.1 Organoleptic measurements

One trained and calibrated examiner assessed the mouth odor for all subjects in this study. Subjects were asked to keep their lips closed tightly for 3 min and then lightly exhaled air from their mouth through a paper tube. The judge rated oral malodor on a 0–5 scale, where a score of 0 represented no odor, 1 = *barely noticeable odor*, 2 = *slight but clearly noticeable malodor*, 3 = *moderate malodor*, 4 = *strong malodor*, and 5 = *extremely strong malodor*, close to saturation (Greenman et al., 2004; Rosenberg & McCulloch, 1992). Subjects were diagnosed as having oral malodor in this study when their organoleptic score was 2 or greater (Murata, Yamaga, Iida, Miyazaki, & Yaegaki, 2002).

#### 2.4.2 VSC measurements

The subjects were required to breathe through their nose for 3 min. A 1‐ml syringe was placed in the mouth, a volume of 1 ml drawn, and then 0.5 ml removed; the remaining 0.5 ml of gas was injected into the receiver of the machine. Hydrogen sulphide (H_2_S) and methyl mercaptan (CH_3_SH) gases were analyzed, and the concentration displayed (ng/10 ml). A previous study suggested that the threshold levels of oral malodor by VSC concentrations were H_2_S of 1.5 ng/10 ml or higher and CH_3_SH of 0.5 ng/10 ml or higher (Tonzetich, 1977).

### 2.5 Oral status assessment

Oral status assessment for all participants was conducted by one trained dentist who was not the examiner for organoleptic measurement. Plaque index (PI) and gingival index (GI) were assessed using the method of Loe and Silness (Loe, 1967), and bleeding on probing (BOP) was evaluated at four sites (distal, buccal, mesial, and lingual) on all teeth except for third molars. Evaluation of tongue coating was based on the criteria of Winkel (Winkel, Roldán, Van Winkelhoff, Herrera, & Sanz, 2003). The subject was asked to put his/her tongue out as far as possible. The back of the tongue from the V‐shaped sulcus to the tip was divided into six sections: three sections on the front half and three sections on the back half. Scores for a section were recorded on a scale from 0 to 2 by visual inspection, where a score of 0 = *no coating*, 1 = *light coating*, and 2 = *severe coating*. Tongue‐coating score was obtained by the addition of all six scores, range 0–12.

Whole saliva was collected by asking subjects to spit all the saliva in their mouth into a plastic cup for 5 min and was measured in milliter per minute. The pH of resting saliva was determined by a pH paper test (Saliva‐Check Buffer Kit, GC, Japan). Measurement of pH and flow rate of saliva were performed by one 6‐year dental student.

### 2.6 Salivary bacteria assessment

Resting saliva samples of all participants were collected in sterile plastic tubes and delivered to the laboratory within 4 hr. A multiplex real‐time polymerase chain reaction (PCR) assay was processed in this study for detection and determination of bacterial species *A*. *actinomycetemcomitans*, *F*. *nucleatum*, *P*. *gingivalis*, S. moorei, *S*. *salivarius*, *T*. *denticola*, and *T*. *forsythia* in the resting saliva of all subjects at baseline and after 2 weeks.

The multiplex real‐time PCR technique was conducted with primers and TaqMan probes specific for the agents' *A*. *actinomycetemcomitans*, *F*. *nucleatum*, *P*. g*ingivalis*, S. moorei, *S*. *salivarius*, *T*. *denticola*, and *T*. *forsythia*. Multiplex real‐time PCRs were prepared from the Qiagen's HotStarTaq Master Mix 2x with 10‐pM primers and 2‐pM TaqMan probe primers for each reaction volume; then, 5‐μl of extracted DNA volume was added to each reaction volume. Two multiplex real‐time PCR mixes were prepared. Multiplex real‐time PCR mix I was used to detect and quantify *A*. *actinomycetemcomitans*, *T*. *denticola*, and *F*. *nucleatu* m. Multiplex real‐time PCR mix II was used to detect and quantify *P*. *gingivalis*, *T*. *forsythia*, *S*. *salivarius*, and S. moorei (Coffey, Mydah Choudhry, & Shlossman, 2016; Corless et al., 2000; Rolph et al., 2001; Srinivasan, Gertz, Shewmaker, et al., 2012). The sequences of primers and TaqMan probes are presented in Table 1.

**Table 1:** Sequences of primers and TaqMan probes used in polymerase chain reaction (PCR)

Sequences
Multiplex real‐time PCR I
*Aggregatibacter actinomycetemcomitans*
Primer F GCGAAACGAAGAGAAGCAAG
Primer R CCTACCCAACAGGCGTATCA
Probe FAM‐ATTCCCAACCGCACTT‐BHQ1
*Treponema denticola*
Primer F GTTGTTCGGAATTATTGG
Primer R GATTCAAGTCAAGCAGTA
Probe Texas Red‐TCACACCAGGCTTACC‐BHQ
*Fusobacterium nucleatum*
Primer F GGCTTCCCCATCGGCATTCC
Primer R AATGCAGGGCTCAACTCTGT
Probe CY5‐TCCGCTTACCTCTCCAG‐BHQ3
Multiplex real‐time PCR II
*Porphyromonas gingivalis*
Primer F CTGCGTATCCGACATATC
Primer R GGTACTGGTTCACTATCG
Probe FAM‐ACCATAGACGACGGAGCACC‐BHQ1
*Tannerella forsythia*
Primer F GAGGTTGTGGAAGGTATG
Primer R GTAGATCAGAATGTACGGATT
Probe Texas Red‐TCTCCGCTTATTTCGTGAC‐BHQ2
*Streptococcus salivarius*
Primer F CACGCCATGCTGGAAGTG
Primer R GCGATGAGCCAAGCTGAAG
Probe CY5‐TTAGCTGCTGCGTAGACTTCGTCT‐BHQ3
*Solobacterium moorei*
Primer F CGGGTGAGTAATACATAAGCAAC
Primer R ACCTTTAATAACCAGAAGATGCCT
Probe HEX‐TGGGATAATCTCTGGAAACGGGGACT‐BHQ1

After preparation, multiplex real‐time PCR mixes were immediately put into a Bio‐Rad real‐time PCR system (CFX 96), and a preheat program was run at 95°C for 15 min to activate the hot‐start Taq polymerase; then, 40 cycles were run, each cycle consisting of two steps, at 94°C for 15 s and 60°C for 30 s. Real‐time results were determined by four color channels: FAM, HEX, Texas Red, and CY5. To control the sensitivity of the PCR mix, positive and negative controls were always run simultaneously. The positive control was the specific synthesized oligosaccharides for primers and TaqMan probes, whereas TE1X was the negative control. In addition, standard curves were made by amplifying the number of copies of specific oligos for the target agent, to calculate the number of bacterial copies detected. After completing the PCR, the cycle threshold (Ct) value of the unknown sample was compared with the standard curve to determine the quantity of targets in the unknown samples.

### 2.7 Experimental assessment

Subjects were evaluated for organoleptic scores and the amount of VSCs at baseline (T0), after 12 hr (T1) and after 2 weeks (T2) of using mouthwash. PI, GI, BOP, tongue‐coating score, salivary pH and flow rate, and the number of salivary bacteria of species' *A*. *actinomycetemcomitans*, *F*. *nucleatum*, *P*. *gingivalis*, S. moorei, *S*. *salivarius*, *T*. *denticola*, and *T*. *forsythia* were evaluated at baseline (T0) and after 2 weeks of using mouthwash (T2).

### 2.8 Statistical analysis

Statistical analysis was performed using the Statistical Package for the Social Sciences software version 22.0 (International Business Machines, Japan). Differences in variables between the two mouthwashes at each examination point were analyzed with the Student's *t* test or Mann–Whitney *U* test. Differences in variables between “before and after” rinsing in each group were analyzed with paired *t* tests. For all analyses, a 5% significance level was used.

## 3 RESULTS

### 3.1 Characteristics of subjects

Accept one subject did not follow full protocol because of his own inconvenience, a total of 39 subjects, 19 men and 20 women, aged from 19 to 23 years, completed the study. They did not suffer from any negative side effect of the mouthwashes. There were 17 subjects initially using the experimental mouthwash then control mouthwash; 22 subjects used the mouthwashes in the reverse order. There were no statistically significant differences for oral malodor, periodontal parameters, salivary pH and flow rate, tongue coating, or number of salivary bacteria between the two groups at the beginning of the study (T0).

### 3.2 Oral malodor

#### 3.2.1 Organoleptic score

A statistically significant reduction in organoleptic scores occurred in the experimental group, from 2.67 ± 1.00 at baseline to 1.64 ± 1.11 after 12 hr and to 0.95 ± 0.86 after 2 weeks (*p* < 0.001). The control group, on the other hand, showed no significant difference in organoleptic scores after 12 hr and after 2 weeks when compared with the baseline scores (Table 2).

**Table 2:** Organoleptic measurement scores at baseline (T0), after 12 hr (T1) and after 2 weeks (T2)

	Experimental group	Control group	*p* value
T0	2.67 ± 1.00	2.82 ± 0.72	0.13^b^
T1	1.64 ± 1.11	2.64 ± 0.87	<0.001^b^
*p* value	<0.001^a^	0.09^a^	
T2	0.95 ± 0.86	2.61 ± 1.01	<0.001^b^
*p* value	<0.001^a^	0.07^a^	

*Note*. Data are presented as means ± *SD*.

a Wilcoxon signed‐rank test.

b Mann–Whitney *U* test; significance at *p* < 0.05.

#### 3.2.2 VSC concentration

In the experimental group, after 12 hr (T1) and after 2 weeks (T2) of rinsing, the concentration of H_2_S showed a statistically significant decrease to 3.69 and 1.07 ng/10 ml, respectively, compared with the baseline (T0; *p* < 0.001). In the control group, after 12 hr (T1) and after 2 weeks (T2) of rinsing, the concentration of H_2_S showed a statistically significant decrease to 5.71 and 5.61 ng/10 ml, respectively, with no significant difference from the baseline (T0). The experimental mouthwash showed a significantly greater reduction in H_2_S level at T1 and T2 (2.31 ± 2.08 and 4.94 ± 5.59 ng/10 mL, respectively) when compared to the control mouthwash (0.27 ± 1.05 and 0.37 ± 1.22 ng/10 ml, respectively; *p* < 0.05; Table 3).

**Table 3:** Concentration of volatile sulphur compounds (ng/10 ml) at baseline (T0), after 12 hr (T1) and after 2 weeks (T2)

		Experimental group	Control group	*p* value
H_2_S	T0	6.00 ± 5.90	5.98 ± 5.34	0.94^b^
	T1	3.69 ± 4.78	5.71 ± 5.21	<0.001^b^
	*p* value	<0.001^a^	0.11^a^	
	T2	1.07 ± 0.84	5.61 ± 4.87	<0.001^b^
	*p* value	<0.001^a^	0.06^a^	
	ΔT1–T0	−2.31 ± 2.08	−0.27 ± 1.05	<0.001^b^
	ΔT2–T0	−4.94 ± 5.59	−0.37 ± 1.22	<0.001^b^
	*p* value	0.001^a^	0.64^a^	
CH_3_SH	T0	2.55 ± 2.07	2.65 ± 2.19	0.85^b^
	T1	1.53 ± 1.92	2.43 ± 1.91	<0.001^b^
	*p* value	<0.001^a^	0.10^a^	
	T2	0.63 ± 0.69	2.38 ± 1.97	<0.001^b^
	*p* value	<0.001^a^	0.12^a^	
	ΔT1–T0	−1.02 ± 1.09	−0.22 ± 0.83	<0.001^b^
	ΔT2–T0	−1.92 ± 1.81	−0.27 ± 1.08	<0.001^b^
	*p* value	0.002^a^	0.58^a^	

*Note*. Data are presented as means ± *SD*.

a Paired *t* test.

b Independent *t* test; significance at *p* < 0.05.

In the experimental group, after 12 hr (T1) and after 2 weeks (T2) of rinsing, the concentration of CH_3_SH showed a statistically significant decrease to 1.53 and 0.63 ng/10 ml, respectively, compared with the baseline (T0; *p* < 0.001). In the control group, after 12 hr (T1) and after 2 weeks (T2) of rinsing, the concentration of CH_3_SH showed a statistically significant decrease to 2.43 and 2.38 ng/10 ml, respectively, with no significant difference from the baseline (T0). The experimental mouthwash showed a significantly greater reduction in CH_3_SH level at T1 and T2 (1.02 ± 1.09 and 1.92 ± 1.81 ng/10 ml, respectively) when compared with the control mouthwash (0.22 ± 0.83 and 0.27 ± 1.08 ng/10 ml, respectively; *p* < 0.05; Table 3).

### 3.3 Periodontal parameters, tongue‐coating scores, and salivary parameters

After using the experimental mouthwash for 2 weeks, a statistically significant reduction in PI was shown compared with the baseline (*p* < 0.05). After use of the control mouthwash for 2 weeks, on the other hand, no statistically significant decrease was observed compared with before rinsing. The mean PI of the experimental group was significantly lower than the mean of the control group after 2 weeks (*p* < 0.05). From baseline to T2, the experimental mouthwash showed a significantly greater reduction in PI (ΔT2–T0 = 0.26 ± 0.59) when compared with the control (ΔT2–T0 = 0.01 ± 0.42; *p* = 0.04). Mean GI and the percentage of BOP after 2 weeks had no statistically significant difference with the values before rinsing, in either the experimental or control group. In the experimental group, after 2 weeks, a statistically significant reduction in tongue‐coating score was evident compared with the baseline (*p* < 0.001), whereas there was no significant difference in the tongue‐coating score of the control group after 2 weeks compared with the baseline. From baseline to T2, the experimental mouthwash showed a significantly greater reduction in tongue‐coating score (ΔT2–T0 = 2.11 ± 1.45) when compared with the control (ΔT2–T0 = 0.79 ± 1.13; *p* = 0.02). There was no statistically significant difference between salivary flow rate and pH for either the experimental or control group after 2 weeks compared with before rinsing (Table 4).

**Table 4:** Periodontal parameters, tongue‐coating scores, and salivary parameters at baseline (T0) and after 2 weeks (T2)

		Experimental group	Control group	*p* value
PI	T0	1.67 ± 0.57	1.56 ± 0.40	0.33^b^
	T2	1.41 ± 0.30	1.54 ± 0.29	0.03^b^
	*p* value	0.01^a^	0.84^a^	
	ΔT2–T0	−0.26 ± 0.59	−0.01 ± 0.42	0.04^b^
GI	T0	1.87 ± 1.10	1.67 ± 0.71	0.38^b^
	T2	1.61 ± 0.81	1.54 ± 0.54	0.26^b^
	*p* value	0.05^a^	0.18^a^	
	ΔT2–T0	−0.26 ± 0.77	−0.13 ± 0.62	0.47^b^
BOP	T0	8.42 ± 1.76	7.65 ± 2.12	0.09^b^
	T2	7.64 ± 1.68	7.53 ± 2.01	0.80^b^
	*p* value	0.08^a^	0.74^a^	
	ΔT2–T0	−0.77 ± 2.62	−0.11 ± 2.12	0.22^b^
Tongue‐coating score	T0	9.32 ± 1.44	9.43 ± 1.14	0.12^b^
	T2	7.21 ± 1.13	8.64 ± 1.99	<0.001^b^
	*p* value	<0.001^a^	0.06^a^	
	ΔT2–T0	−2.11 ± 1.45	−0.79 ± 1.13	0.02^b^
Salivary flow rate, ml/min	T0	0.35 ± 0.03	0.35 ± 0.04	0.88^b^
	T2	0.36 ± 0.04	0.34 ± 0.04	0.47^b^
	*p* value	0.14^a^	0.94^a^	
	ΔT2–T0	0.01 ± 0.03	0.00 ± 0.03	0.24^b^
Salivary pH	T0	7.17 ± 1.32	7.31 ± 1.46	0.24^b^
	T2	7.23 ± 1.26	7.31 ± 1.35	0.15^b^
	*p* value	0.42^a^	0.92^a^	
	ΔT2–T0	0.06 ± 0.02	0.00 ± 0.01	0.21^b^

*Note*. BOP: bleeding on probing; GI: gingival index; PI: plaque index. Data are presented as means ± *SD*.

a Paired *t* test.

b Independent *t* test; significance at *p* < 0.05.

### 3.4 Amount of salivary bacteria *A*. *actinomycetemcomitans*, *F*. *nucleatum*, *P*. *gingivalis*, S. moorei, *S*. *salivarius*, *T*. *denticola*, and *T*. *forsythia* (log_10_[copies/10 μl])

After 2 weeks, in the experimental group, the counts of *F*. *nucleatum*, S. moorei, *T*. *denticola*, and *T*. *forsythia* were significantly reduced compared with the baseline (*p* < 0.001). The control mouthwash group after 2 weeks, on the other hand, showed no statistically significant reduction compared with the baseline. There was a statistically significant reduction in the counts of *F*. *nucleatum*, S. moorei, *T*. *denticola*, and *T*. *forsythia* between the experimental and control groups after 2 weeks (*p* < 0.05). There were no significant changes in the amounts of *A*. *actinomycetemcomitans*, *P*. *gingivalis*, or *S*. *salivarius* in either group compared with the baseline. There were no differences in the levels of *A*. *actinomycetemcomitans*, *P. gingivalis*, or *S. salivarius* between the experimental and control groups after 2 weeks (Table 5).

**Table 5:** Amount of *Aggregatibacter actinomycetemcomitans*, *Fusobacterium nucleatum*, *Porphyromonas gingivalis*, Solobacterium moorei, *Streptococcus salivarius*, *Treponema denticola*, and *Tannerella forsythia* (log_10_[copies/10 μl]) in saliva in the experimental and control groups at baseline (T0) and after 2 weeks (T2)

		Experimental group	Control group	*p* value
*A*. *actinomycetemcomitans*	T0	1.62 ± 2.54	1.67 ± 2.31	0.93^b^
	T2	1.36 ± 2.42	1.17 ± 2.24	0.72^b^
	*p* value	0.41^a^	0.11^a^	
	ΔT2–T0	−0.26 ± 1.95	−0.49 ± 1.89	0.57^b^
*F*. *nucleatum*	T0	7.27 ± 0.57	7.15 ± 1.40	0.57^b^
	T2	6.65 ± 0.86	7.10 ± 1.77	0.15^b^
	*p* value	<0.001^a^	0.79^a^	
	ΔT2–T0	−0.63 ± 0.79	−0.05 ± 1.18	0.03^b^
*P*. *gingivalis*	T0	2.54 ± 2.96	2.16 ± 2.98	0.39^b^
	T2	2.64 ± 2.95	2.24 ± 2.79	0.26^b^
	*p* value	0.77^a^	0.65^a^	
	ΔT2–T0	0.11 ± 1.46	0.08 ± 1.72	0.1^b^
S. moorei	T0	6.92 ± 1.43	6.9 ± 1.00	0.79^b^
	T2	5.58 ± 2.14	6.64 ± 1.44	0.15^b^
	*p* value	<0.001^a^	0.16^a^	
	ΔT2–T0	−1.35 ± 2.02	−0.35 ± 1.54	0.01^b^
*S*. *salivarius*	T0	6.32 ± 2.09	6.52 ± 1.49	0.59
	T2	6.08 ± 2.11	6.42 ± 1.38	0.38
	*p* value	0.55	0.37	
	ΔT2–T0	−0.24 ± 1.65	−0.09 ± 0.98	0.65
*T*. *denticola*	T0	3.38 ± 3.07	3.71 ± 3.04	0.12^b^
	T2	2.65 ± 2.98	3.57 ± 2.94	0.04^b^
	*p* value	0.01^a^	0.54^a^	
	ΔT2–T0	−0.73 ± 1.63	−0.14 ± 1.37	0.01^b^
*T*. *forsythia*	T0	5.83 ± 2.08	5.89 ± 1.67	0.85^b^
	T2	5.45 ± 2.03	5.87 ± 1.61	0.08^b^
	*p* value	<0.001^a^	0.91^a^	
	ΔT2–T0	−0.39 ± 0.60	−0.01 ± 0.66	0.04^b^

*Note*. Data are presented as means ± *SD*.

a Paired *t* test.

b Independent *t* test; significance at *p* < 0.05.

## 4 DISCUSSION

In this randomized clinical trial, two mouthwashes, one with 0.1% ClO_2_ and one of 0.9% sodium chloride solution, were compared to investigate the malodor, periodontal parameter, tongue coating, and salivary bacteria‐reducing effects of ClO_2_. The results of the present investigation demonstrate the beneficial effects on oral malodor, plaque, tongue‐coating accumulation, and salivary bacteria of using a mouthwash containing 0.1% ClO_2_ for 2 weeks in halitosis subjects when compared with the control mouthwash.

A randomized crossover design in this study helps to reduce the influence of confounding covariates because each subject serves as his or her own control. Four weeks of washout in our study was longer than that in previous studies about ClO_2_ mouthwash, such as the studies of Shinada et al. (2010; Shinada et al., 2010) and of Soares et al. (2013; Soares et al., 2013). With this period of washout, the oral condition returned to baseline after 4 weeks, and there were no significant differences in any clinical parameters between the two groups at baseline in either the first or second stage. Similarly, there were no significant differences between the baselines in the first and second stages of each mouthwash after the 4‐week washout period.

ClO_2_ is a free radical that is soluble in water and stable for a long period of time without exposure to light. Previous research has shown that ClO_2_ and chlorite anion are effective antimicrobial agents against many species of bacteria (Grootveld et al., 2001). When existing in the mouth, ClO_2_ reacts with amino acids found in saliva, such as pyruvate, methionine, trimethylamine, tyrosine, and glycine, which are nutrients for odor‐causing bacteria. Therefore, ClO_2_ may inhibit malodor production by interrupting the growth of bacteria through the nutrient pathway. In addition, thanks to its good solubility in water; ClO_2_ can penetrate biofilm easily and increase antibacterial efficiency (Grootveld et al., 2001).

VSC levels are closely related to the level of oral malodor. Free chloride ions chemically oxidize odorous substances, such as VSCs, to odorless chloride forms. In a study, Lynch et al. (1997) used high‐resolution spectroscopy to determine that oxidation also consumed the amino acids cysteine and methionine, precursors of VSCs (Lynch et al., 1997). On the other hand, ClO_2_ can help to create an oxygen‐rich environment in the oral cavity, limiting the growth of malodor‐associated bacteria, especially anaerobic species. In this study, we found that rinsing with mouthwash containing 0.1% ClO_2_ significantly reduced VSCs (H_2_S, CH_3_SH) after 12 hr compared with the baseline, and this reduction was more noticeable after 2 weeks of use. The results also showed a significant improvement in organoleptic score from the baseline after 12 hr and after 2 weeks. These findings are consistent with those of previous studies demonstrating the efficacy of ClO_2_‐containing mouthwash in decreasing VSCs and controlling bad breath. A randomized, crossover clinical trial by Peruzzo et al. (2007) demonstrated that after 4 days of not performing any oral hygiene but using mouthwash, VSC concentration was not significantly different to the baseline in the ClO_2_ mouthwash group, while it doubled in the placebo group (Peruzzo et al., 2007). Shinada et al. (2008) reported that 0.1% ClO_2_ mouthwash helped to reduce organoleptic measurement scores and VSC amount significantly, and this effect remained for up to 4 hr after rinsing (Shinada et al., 2008). Neetha et al. (2013) showed that 0.1% ClO_2_ mouth rinse can prevent the formation of VSCs equivalent to 0.2% chlorhexidine (CHX) mouth rinse after using for 7 days.(Shetty, David, Kamala, & Shenoy, 2013).

In the ClO_2_ group, 12 hr after rinsing, we found that the concentrations of H_2_S and CH_3_SH were significantly reduced but still higher than the olfactory threshold levels for H_2_S (1.5 ng/10 ml) and CH_3_SH (0.5 ng/10 ml). After 2 weeks, the H_2_S concentration was lower than the diagnosis threshold for H_2_S, but the CH_3_SH level still remained higher than the threshold for CH_3_SH. Contrary to our results, Shinada et al. showed that rinsing with 0.1% ClO_2_ for 7 days reduced the concentration of CH_3_SH far below the olfactory threshold level (Shinada et al., 2010). This difference might be due to the fact that the subjects selected in our study had genuine oral malodor, with much higher concentrations of H_2_S and CH_3_SH in mouth air than healthy participants in other studies. This result of our study showed that mouthwash containing 0.1% ClO_2_ was more effective in reducing oral malodor due to H_2_S than due to CH_3_SH after 2 weeks of use.

This study showed that using 0.1% ClO_2_ mouthwash for 2 weeks caused a statistically significant decreased in plaque accumulation, whereas the gingivitis and bleeding indices also tended to be reduced but not by a statistically significant amount. Some research has reported that ClO_2_ mouthwash has an inhibitory effect on plaque growth but not sufficient to effectively reduce gingivitis (Shetty et al., 2013; Shinada et al., 2008). On the other hand, a study by Sravan et al. (2015) on orthodontic patients found that ClO_2_ oral rinses decreased both PI and GI equivalents to use of 0.12% CHX (Sravan, Shashidhar, & Arun, 2015). In our study, compared with saline, ClO_2_ mouthwash prevented the formation of plaque more effectively. The mechanism of this process may be due to the fact that the antimicrobial property of ClO_2_ alters the oral environment and prevents bacteria from adhering to dental surfaces, hence inhibiting the formation of plaque.

In the present study, after 2 weeks, tongue‐coating status was clearly affected by the 0.1% ClO_2_ mouthwash, as demonstrated by the significant reductions in mean tongue‐coating score in the experimental group. The “rinsing and gargling” fashion of using mouthwash in this study might also have an effect on the degree of tongue coating. However, in our study, the control group did not have a statistically significant decrease in tongue‐coating score after 2 weeks. Therefore, we found that the mouthwash containing 0.1% ClO_2_ was effective in reducing tongue plaque after 2 weeks of use. A reduction in tongue plaque due to ClO_2_ was also observed in the study by Shinada et al (Rosenberg & McCulloch, 1992). Another study by Yadav et al. (2015) demonstrated that after 5 days of use, 0.1% ClO_2_ mouthwash effectively decreased tongue coating equivalently to CHX 0.2% (Yadav, Kini, & Padhye, 2015). Tongue coating has been shown to be associated with malodor levels and plays the most important role in the production of VSCs (Pham, 2013; Pham, Ueno, Shinada, & Kawaguchi, 2012; Pham et al., 2011; Pham et al., 2010). The results of our study suggest that these limitations can be overcome, to support tongue‐coating control, with mouthwash containing antibacterial agents such as ClO_2_.

High salivary pH and low salivary flow rate are among the risk factors for halitosis. A low salivary flow rate may lead to a lack of the washing effect of saliva, making it easier for odorous compounds to evaporate from oral surfaces and become more noticeable in the breath (Kleinberg, Wolff, & Codipilly, 2002). After 2 weeks, the study found no change in pH or resting salivary flow rate in both experimental and control groups, which means ClO_2_‐containing mouthwash did not significantly alter pH or salivary flow rate.

A number of studies recently have shown new interest in the correlation between periodontal bacteria and oral malodor (Nakano et al., 2002; Pham, Ueno, Shinada, & Kawaguchi, 2013; Yasukawa, Ohmori, & Sato, 2010). In the present study, we assessed changes in five strains of periodontopathic bacteria, *A*. *actinomycetemcomitans*, *F*. *nucleatum*, *P*. *gingivalis*, *T*. *forsythia*, and *T*. *denticola*, after using mouthwash. After 2 weeks of rinsing, our study demonstrated that 0.1% ClO_2_ mouthwash had significant antibacterial activity against *F*. *nucleatum* and two species belonging to the red complex, *T*. *forsythia* and *T*. *denticola*, whereas no significant changes were made to *A*. *actinomycetemcomitans* or *P*. *gingivalis*. In contrast to our results, Shinada et al. (2008) found that after 7 days of using 0.1% ClO_2_ mouthwash, only *F*. *nucleatum* was significantly decreased, whereas there were no changes in *T*. *denticola*, *P*. g*ingivalis*, or *T*. *forsythia* counts (Shinada et al., 2008). *A*. *actinomycetemcomitans* does not produce hydrogen sulfide and methyl mercaptan. However, the possible role of *A*. *actinomycetemcomitans* in hydrogen sulfide production from *T*. *denticola* has been reported (Chu et al., 2009). As previously mentioned, these conflicts may be explained by the differences in microbial flora characteristics between nonhalitosis and halitosis participants in the two studies. In addition, using an antimicrobial mouthwash over a longer period of time may provide more thorough antimicrobial efficacy.

*F*. *nucleatum*, *T*. *forsythia*, and *T*. *denticola* have been known to be involved in the production of oral malodor (Nakano et al., 2002; Yasukawa et al., 2010). The decreasing amounts of these organisms might correspond with the reduction in organoleptic scores and VSC levels following the use of the 0.1% ClO_2_ mouthwash. Yasukawa et al. (2010) reported that subjects who had tongue plaque positive for *F*. *nucleatum* and *T*. *denticola* exhibited a greater tendency to have physiologic halitosis, and the presence of *T*. *forsythia* may also indicate an increased risk for halitosis (Yasukawa et al., 2010). *F*. *nucleatum* has been proposed as a “bridge” bacterium, one which helps to facilitate favorable conditions for other halitosis‐causing bacteria, especially *T*. *forsythia*. *T*. *forsythia* is also thought to be associated with the production of CH_3_SH, with significantly higher CH_3_SH levels observed in halitosis patients than in healthy individuals (Awano et al., 2002). In an in vitro study, *T*. *denticola*, *P*. *gingivalis*, and *T*. *forsythia* appeared to produce the most abundant VSCs, which play an important role in the pathogenesis of halitosis (Persson et al., 1990). In this study, however, we were unable to find a significant reduction in *A*. *actinomycetemcomitans* or *P*. *gingivalis* bacterial load after using the ClO_2_ mouthwash. An investigation by Nakano et al. suggested that *A*. *actinomycetemcomitans* does not form methyl mercaptan and may be less likely to produce VSCs than *T*. *denticola* or *P*. gingivalis (Nakano et al., 2002). Although *P*. *gingivalis* produces a large amount of VSCs in vitro (Persson et al., 1990), there may be more important factors affecting halitosis. Therefore, oral malodor parameters showed improvements after 2 weeks of using 0.1% ClO_2_ although *A*. *actinomycetemcomitans* and *P*. *gingivalis* were not reduced.

Previous studies have reported that a greater amount and more frequent presence of S. moorei are found in patients with oral malodor but not in control subjects (Haraszthy et al., 2008). Recently, Vancauwenberghe et al. (2013) reported a significant correlation between S. moorei, tongue coating, and total VSCs (Vancauwenberghe et al., 2013). Stephen et al. (2014) demonstrated that when cultured in vitro, S. moorei produces H_2_S directly from cysteine in quantities two to three times greater than *P*. *gingivalis* (Stephen et al., 2014). This correlation can be explained by the fact that S. moorei is not only capable of producing VSCs, especially H_2_S, but also possesses the enzyme β‐galactosidase, which can cut glycoprotein chains from saliva into substrates for Gram‐negative microorganisms to produce odorous compounds (Haraszthy et al., 2008). In this study, we showed that ClO_2_ mouthwash could reduce the number of S. moorei. Considering this result, we supposed that S. moorei may interact with *F*. *nucleatum*, *T*. *forsythia*, and *T*. *denticola* in different steps of VSC production, with S. moorei (Gram positive) being responsible for deglycosylation, and *F*. *nucleatum*, *T*. *forsythia*, and *T*. *denticola* (Gram negative) degrading protein substrates, resulting in a decrease in oral malodor and the amount of VSCs. However, further microbiological studies of bacterial communities are required to understand these interactions.

*S*. *salivarius* displays a tendency to adhere to oral epithelial cells and is one of the earliest microorganisms, which colonizes oral mucosa surfaces (Kazor, Mitchell, Lee, Stokes, & Loesche, 2003). *S*. *salivarius* represents a high percentage of the total facultative *Streptococci* in samples from the tongue and buccal mucosa of adults (Burton, Chilcott, & Tagg, 2005). An in vitro study suggested that *S*. *salivarius* is also a bacterial strain that promotes the breakdown of salivary components and is associated with malodor formation (Sterer & Rosenberg, 2006). However, Yoshida et al. (2003) supposed that this specie also has only a restricted ability to produce VSCs and is unlikely to contribute significantly to oral malodor (Yoshida, Negishi, Amano, Oho, & Nakano, 2003). Thus far, there has been no report about the effect of ClO_2_ mouthwash on S. moorei nor on *S*. *salivarius*. In this study, we demonstrated that 2 weeks of rinsing with ClO_2_ mouthwash reduced S. moorei but not *S*. *salivarius*.

This study has some limitations. Our study has investigated the 2‐week‐term effects of mouthwash containing 0.1% ClO_2_ on oral malodor treatment in a group of students. Furthermore, a different population and/or different oral conditions may have the different outcomes. Future research involving the longer term evaluation of oral malodor related outcomes in different types of populations are needed. In addition, comparative efficacy studies need to be performed against the known effective mouth rinses containing CHX should be conducted.

## 5 CONCLUSIONS

The results show that a 0.1% ClO_2_ mouthwash significantly reduces oral malodor and the concentrations of H_2_S and CH_3_SH after 12 hr and after 2 weeks. Moreover, this mouthwash is effective in decreasing PI, tongue coating, and the amounts of Gram‐positive and Gram‐negative bacteria such as *F*. *nucleatum*, S. moorei, *T*. *denticola*, and *T*. *forsythia* in saliva. From our results, ClO_2_ is a promising agent in the treatment and management of bad breath.

## 6 CLINICAL RELEVANCE

### 6.1 Scientific rationale for study

Very few clinical trials have shown the effect of mouthwash containing ClO_2_ on oral malodor, salivary pH and flow rate, Gram‐negative and Gram‐positive bacteria, and periodontal status. There have been some studies investigating the impact of ClO_2_ mouthwash on oral malodor status; however, most of them were carried out on people with healthy breath, with short‐term treatment, and rarely assessed the microbiology results, especially for Gram‐positive organisms such as *S*. *moorei* and *S*. *salivarius*, which may also be involved in the production of odorous compounds.

### 6.2 Principal findings

Mouthwash containing 0.1% ClO_2_ is effective in reducing oral malodor including organoleptic score and VSCs, dental plaque, tongue‐coating accumulation, and the amounts of *F*. *nucleatum*, S. moorei, *T*. *denticola*, and *T*. *forsythia* in saliva.

### 6.3 Practical implications

Increasing public concern about bad breath leads to frequent use of mouth rinses to prevent and manage halitosis. Our study suggests that mouthwash containing 0.1% ClO_2_ is a promising agent in the treatment and management of bad breath for 2‐week use.

## CONFLICT OF INTEREST

All authors declare that there are no competing financial interests.

## FUNDING INFORMATION

This study was conducted without any financial support.
